# Omental Lymphangioma in Adults—Rare Presentation Report of a Case

**DOI:** 10.1155/2012/629482

**Published:** 2012-11-06

**Authors:** T. Narayana Rao, T. Parvathi, A. Suvarchala

**Affiliations:** ^1^Andhra Medical College, King George Hospital, Visakhapatnam 530002, India; ^2^Division of Infectious Diseases, Andhra Medical College, Andhra Pradesh, Visakhapatnam 530002, India

## Abstract

Lymphangioma is an uncommon benign lesion that usually occurs during childhood. Its occurrence in adults is rare. Its presentation in the abdomen is even rare. This case report describes a case of omental lymphangioma presented as retroperitoneal lump. Subsequent imaging, operative, and histological findings revealed omental lymphangioma. Laparotomy done under general anesthesia, a 10 × 12 cm cystic swelling arising from omentum, identified complete excision of the cyst done and send the specimen for histopathological examination. Biopsy report came as omental lymphangioma. Complete surgical excision is the treatment of choice. Prognosis is excellent and recurrence rate is very low if resection is complete. During two years of followup no recurrence was detected. Omental lymphangioma is very rare presentation among abdominal lymphangiomas specifically in adults. Complete excision is the treatment of choice. Long-term followup is required to detect recurrence.

## 1. Introduction

Lymphangioma is an uncommon benign lesion that is due to result from a developmental failure of lymphatic system/inflammation of lymhatics causing obstruction. Lymphangiomas occur in many anatomical locations. Most cases (95%) are found in the neck and axillary regions, whereas other sites, such as the mesentery, *retroperitoneum,* abdominal viscera, bone, lung, and mediastinum are unusual. Although rare abdominal lymphangiomas are more common in boys than in girls, they usually occur during childhood. They are reported to occur most commonly in the mesentery, followed by omentum, mesocolon, and retroperitoneum. They arise in all age groups and have variable presentation. But they are extremely rare in adult population [1]. Lymphangiomas are thought to result from a developmental failure of lymphatic system. Another possibility includes inflammation of lymphatic system leading to obstruction and subsequent development of lymphangioma. They are often confused with mesenteric cysts that arise from mesothelial, not lymphatic tissue. This differentiation is important because lymphangiomas often behave in an invasive and aggressive manner, whereas mesothelial cysts do not. Despite being difficult to differentiate between imaging studies, they are histologically distinct from one another. Lymphangiomas have an endothelial lining, foam cells, and a wall that contains lymphatic spaces, lymphoid tissues, and smooth muscles. Treatment is surgical excision.

## 2. Case Report

**Figure 1:**
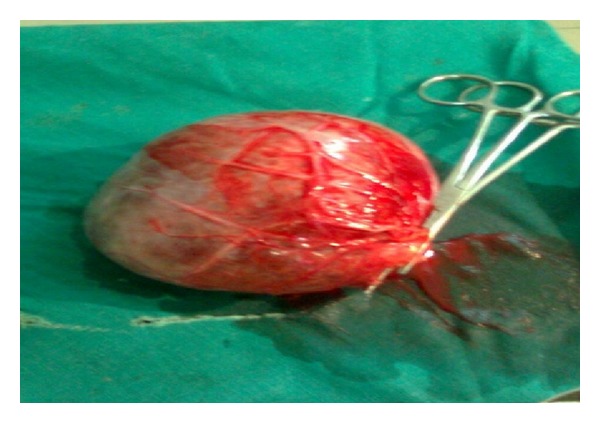
Omental lymphangiomas gross specimen appearance.

A 35-year-old female patient presented with a history of pain abdomen. On examination abdominal lump of size 10 × 12 cm, occupying the right lumbar region extending to umbilical region smooth surface, soft in consistency, as retroperitoneal in position, was identified. Routine blood investigations show no obvious abnormality. As ultrasound abdomen shows 10 × 12 cm cystic anechoic lesion with multiple septations seen suggestive of lymhangioma. CECT abdomen was done showing multiseptate cystic mass with contrast enhancement of cyst walls suggestive of lymphangioma arising from omentum. Laparotomy done, intra operatively a 10 × 12 cm cystic mass occupying umbilical, right lumbar regions extended up to sub hepatic region identified. Complete excision was done. Postoperative period was uneventful. During follow-up period no recurrence was found (Figure 1).

## 3. Discussion

The etiology of lymphangiomas remains unclear. Because lymphangiomas occur mainly in children, the majority of cases are thought to derive from a congenital abnormality of lymphatic system. Clinical presentation can be variable and nonspecific. Acute symptoms include acute abdomen, distension, vomiting, and fever. Chronic symptoms include progressive abdominal distension and pain. Plain radiographs may show noncalcified soft tissue mass, displacement of intestinal loops and small bowel obstruction. Ultrasonography and CECT. are highly sensitive tests that can be used in diagnosis [2]. Sonographically lymphangiomas are anechoic cystic masses that have posterior acoustic enhancement. They can be multilocular with internal septa. Sometimes internal dermis even solid echogenicity with a honey comb pattern, can be demonstrated. Their variable echogenicity is accounted for by the various contents that are possible. CECT. can provide information regarding anatomical location, adjacent organ involvement, size, and complications. On CT scan lymphangiomas are thin walled multiseptated cystic masses. The attenuation of the fluid ranges from that of clear/complicated fluid to that of fat, depending on various contents. The cyst wall and septa can show enhancement after intravenous injection of contrast. Calcification is uncommon [3]. Complications of lymphangioma include hemorrhage, infection, torsion, and small bowel obstruction [4]. Konen et al. suggested that progressive enlargement, multiplication, thickening of septa, and increased echogenicity of cystic fluid are signs which suggest complications that require urgent treatment. For confirmation of diagnosis ultrasound/CECT abdomen should be done. However there can be no specific radiological features to differentiate between mesenteric cyst and lymhangioma hence pathological confirmation is necessary after excision of the lump. Differential diagnosis: Duplication cysts, enteric cysts, pseudocysts, cystic teratoma, cystic leiomyoma, leiomyosarcoma, ovarian cystic masses [5–10]. 

However there can be no specific radiological features to differentiate between these options—histological evaluation may be necessary. Ascites and lymphangioma can also be difficult to differentiate [11]. The presence of septa, compression on adjacent intestinal loops, and lack of fluid in the dependent recess of peritoneum between leaves of small bowel mesentery suggest lymphangioma [12–14]. Malignant degeneration to low grade sarcoma has been reported but is rare. For the present case laparotomy done under general anaesthesia, a 10 × 12 cm cystic swelling arising from omentum, was identified. Complete excision of the cyst was done. Histopathological examination shows features of lymhangioma. During follow-up period of two years no recurrence was identified. 

## 4. Conclusion

Omental lymphangioma is very rare presentation among abdominal lymphangiomas specifically in adults. Complete excision is the treatment of choice. Long-term followup is required to detect recurrence. 

## References

[1] De Perrot M, Rostan O, Morel P, Le Coultre C (1998). Abdominal lymphangioma in adults and children. *British Journal of Surgery*.

[2] Chou YH, Tiu CM, Lui WY, Chang T (1991). Mesenteric and omental cysts: an ultrasonographic and clinical study of 15 patients. *Gastrointestinal Radiology*.

[3] Hancock BJ, St-Vil D, Luks FI, Di Lorenzo M, Blanchard H (1992). Complications of lymphangiomas in children. *Journal of Pediatric Surgery*.

[4] Christensen JA, Fuller JW, Hallock JA, Sherman RT (1975). Mesenteric cysts: a cause of small bowel obstruction in children. *American Surgeon*.

[5] Caropreso PR (1974). Mesenteric cysts: a review. *Archives of Surgery*.

[6] Fowler EF (1961). Primary cysts and tumours of small bowel mesentery. *American Surgeon*.

[7] Takiff H, Calabria R, Yin L, Stabile BE (1985). Mesenteric cysts and intra-abdominal cystic lymphangiomas. *Archives of Surgery*.

[8] Kurtz RJ, Heimann TM, Holt J, Beck AR (1986). Mesenteric and retroperitoneal cysts. *Annals of Surgery*.

[9] Vanek VW, Phillips AK (1984). Retroperitoneal, mesenteric, and omental cysts. *Archives of Surgery*.

[10] De Perrot M, Rostan O, Morel P, Le Coultre C (1998). Abdominal lymphangioma in adults and children. *British Journal of Surgery*.

[11] Lugo-Olivieri CH, Taylor GA (1993). CT differentiation of large abdominal lymphangioma from ascites. *Pediatric Radiology*.

[12] Christensen JA, Fuller JW, Hallock JA, Sherman RT (1975). Mesenteric cysts: a cause of small bowel obstruction in children. *American Surgeon*.

[13] Konen O, Rathaus V, Dlugy E, Kessler A, Shapiro M, Horev G (2002). Childhood abdominal cystic lymphangioma. *Pediatric Radiology*.

[14] Levine C (1989). Primary disorders of the lymphatic vessels—a unified concept. *Journal of Pediatric Surgery*.

